# Supplementary material for: Olive Mill Waste-Water Extract Enriched in Hydroxytyrosol and Tyrosol Modulates Host–Pathogen Interaction in IPEC-J2 Cells
Source: Animals (Basel). 2024 Feb 7;14(4):564. doi: 10.3390/ani14040564 (PMC10886184; doi:10.3390/ani14040564)
Supplement: Supplementary file 1 [file animals-14-00564-s001.zip › animals-2812203-supplementary.pdf]

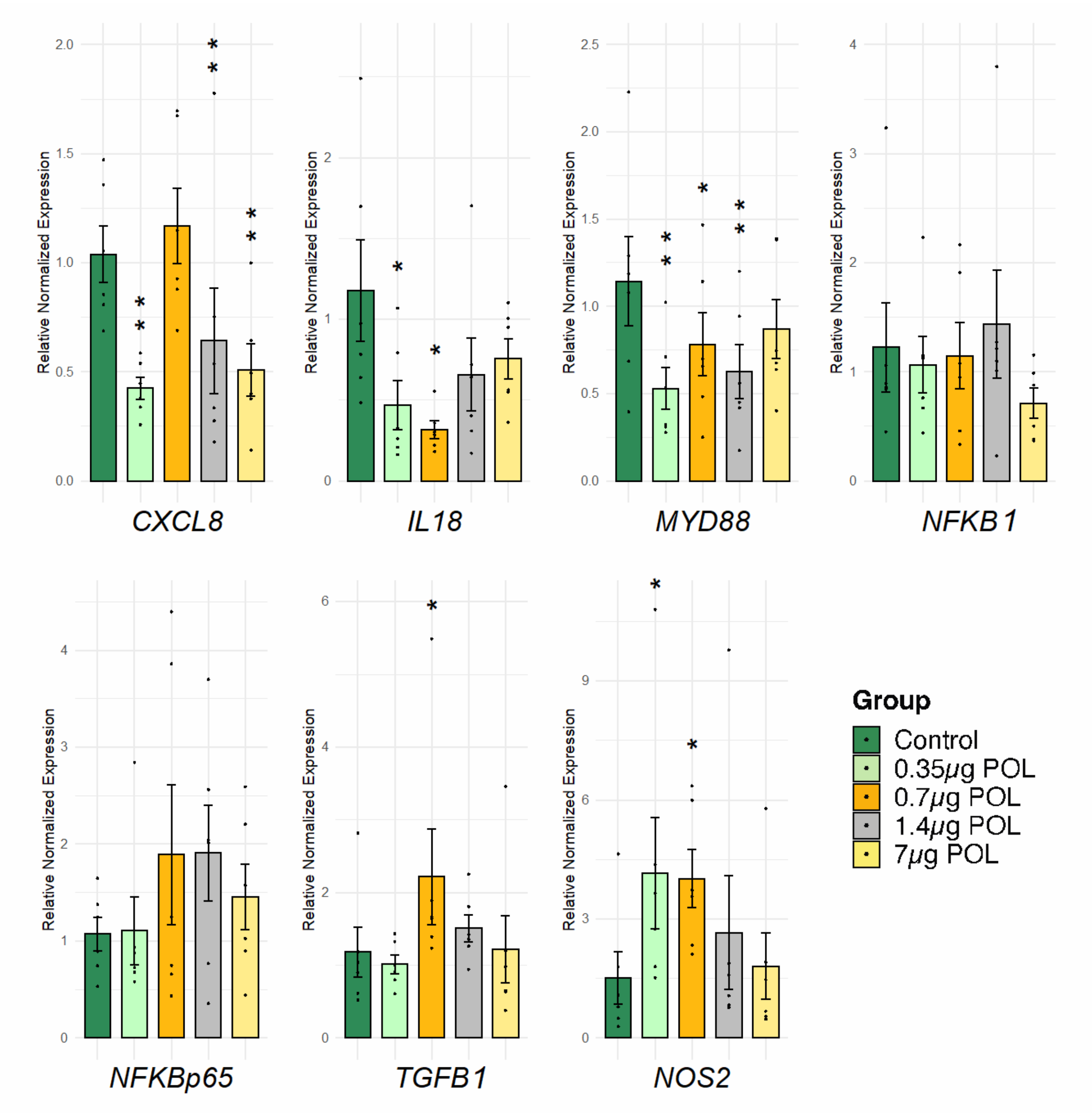

Supplementary Figure S1. Effect of 24h OMWW-extract polyphenols (OMWW-EP) on IPEC-J2 gene expression. The RT-qPCR analysis was performed to evaluate *CXCL8, IL18, MYD88, NFKB1, NFKB/p65, TGFB1, NOS2* gene expression. Data are presented as barr plots displaying mean value of normalized expression and standard error for error bars. Dots represent samples within each group. For each gene, differences between treated polyphenols (0.35 µg POL, 0.7 µg POL, 1.4 µg POL, 7 µg POL) vs untreated (Control) cells were evaluated through one way ANOVA followed by a Dunnett's test or a Kruskal–Wallis test followed by Dunn's multiple comparison test; * p<0.05, ** p<0.01.
